# Impaired Alignment of Bone Matrix Microstructure Associated with Disorganized Osteoblast Arrangement in Malignant Melanoma Metastasis

**DOI:** 10.3390/biom11020131

**Published:** 2021-01-20

**Authors:** Aira Matsugaki, Yumi Kimura, Ryota Watanabe, Fumihito Nakamura, Ryo Takehana, Takayoshi Nakano

**Affiliations:** 1 Division of Materials and Manufacturing Science, Graduate School of Engineering, Osaka University, 2-1 Yamada-oka, Suita, Osaka 565-0871, Japan; yumi.kimura@mat.eng.osaka-u.ac.jp (Y.K.); ryota.watanabe@mat.eng.osaka-u.ac.jp (R.W.); fumihito.nakamura@mat.eng.osaka-u.ac.jp (F.N.); ryo.takehana@mat.eng.osaka-u.ac.jp (R.T.); 2 Teijin Nakashima Medical Co. Ltd., 688-1, Joto-Kitagata, Higashi-ku, Okayama 709-0625, Japan

**Keywords:** malignant melanoma, ex vivo metastasis model, bone tissue microstructure, osteoblast

## Abstract

Malignant melanoma favors spreading to bone, resulting in a weakened bone with a high fracture risk. Here, we revealed the disorganized alignment of apatite crystals in the bone matrix associated with the homing of cancer cells by developing an artificially controlled ex vivo melanoma bone metastasis model. The ex vivo metastasis model reflects the progressive melanoma cell activation in vivo, resulting in decreased bone mineral density and expression of MMP1-positive cells. Moreover, less organized intercellular connections were observed in the neighboring osteoblasts in metastasized bone, indicating the abnormal and randomized organization of bone matrix secreted by disconnected osteoblasts. Our study revealed that the deteriorated microstructure associated with disorganized osteoblast arrangement was a determinant of malignant melanoma-related bone dysfunction.

## 1. Introduction

Malignant melanoma is one of the progressive cancers favoring bone metastasis, and its invasion of bone results in severe skeletal deterioration [1]. Bone metastasis is generally classified into osteoblastic or osteolytic metastasis based on the radiographic diagnosis of the balance between bone formation and resorption [2]. For example, prostate cancer or breast cancer promotes or suppresses bone formation, respectively, thereby influencing the development of bone diseases. On the other hand, malignant melanoma cases present multiple types of pathological defects, and its effects on bone cell activities vary depending on the progressive stages of melanoma [3]. While the bony invasion and homing processes of malignant melanoma cells comprises complicated molecular regulation of cancer-bone crosstalk, the essential biological events triggering bone dysfunction related to melanoma metastasis are poorly understood.

It is increasingly recognized that the mechanical function of intact bone tissue is governed by the anisotropic arrangement of the collagen/apatite matrix structure [4,5], which is derived from bone matrix production by the unidirectionally organized osteoblasts [6,7]. The microstructural orientation of bone tissue varies depending on the anatomical bone portion, which enables the functional adaptation of bone tissue depending on the surrounding mechanical environment [8]. The above microstructural organization of the bone matrix is controlled by unidirectional osteoblast arrangement, which is mediated by molecular interaction through gap junction formation. On the other hand, diseased bone exhibits impaired mechanical functions because of the deteriorated arrangement of crystallographic texture in the bone matrix under impaired control of cellular conditions [9,10]. Particularly, the homing of cancer cells to bone tissue significantly affects bone functionalization via multiple pathways involved in tumor progression [11]. Our recent findings have demonstrated that the disordered collagen/apatite microstructure is a significant contributor to bone dysfunction in metastasized bone, including prostate cancer metastasis [12]. Understanding the significant contributors to cancer-triggered deterioration of the microstructural organization of bone can provide novel therapeutic targets for cancer bone treatment. The ex vivo three-dimensional tumor-generation model is a powerful tool for understanding the biological scenario controlling the interaction between cancer progression and bone tissue organization. In the present study, we constructed a melanoma metastasis model using a harvest device of embryonic bone tissue with a controlled rotating culture of melanoma cells. The developed metastasis system partly mirrors the cellular events mediating melanoma metastasis, and it also enables the control of external factors including the mechanical field for bone tissue organization. The bone tissue cultured with circulating melanoma cells showed a metastasized phenotype with deteriorated alignment of the bone matrix, accompanied by less aligned, disconnected osteoblasts.

## 2. Results

### 2.1. Ex Vivo Melanoma Bone Metastasis Model

**Figure 1:**
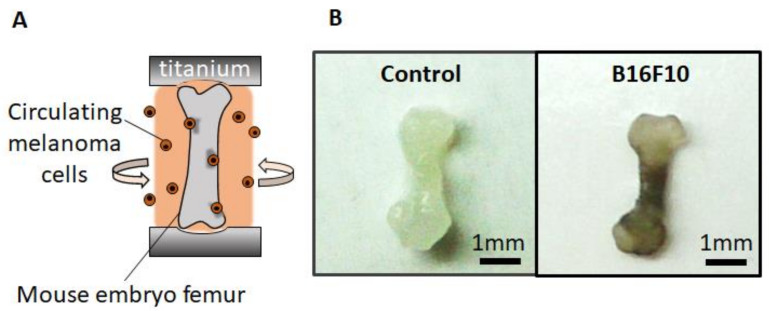
(**A**) Schematic illustration of the ex vivo malignant melanoma metastasis model. (**B**) Appearance of the bone cultured without and with B16F10 melanoma cells.

**Figure 2:**
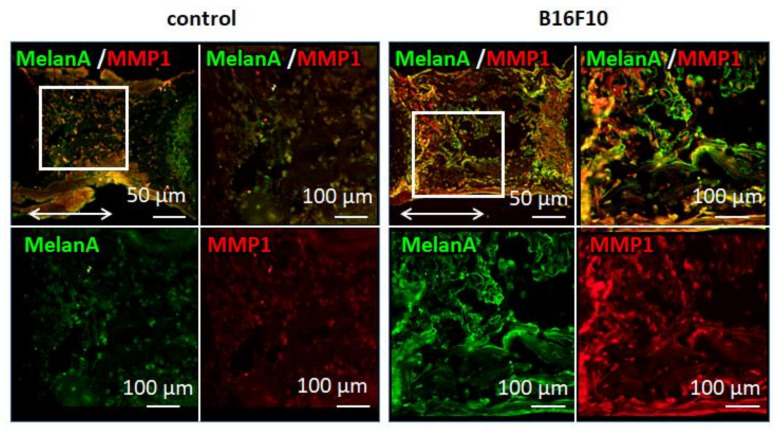
Immunohistochemical analysis of cultured bone without and with B16F10 cancer cells. Green, MelanA; red, MMP1. The double-sided arrows indicate the longitudinal direction of bone. The magnified images of the surrounded area in the upper left side are shown.

The malignant melanoma bone metastasis model was successfully established by the controlled culturing of embryonic bone tissue inside a static uniaxial loading platform. Femurs cocultured with malignant melanoma cells resulted in a blackened appearance, thereby indicating the production of melanin by colonized melanoma cells (Figure 1). Immunohistochemical analysis demonstrated that coculture with malignant melanoma cells induced a high expression of melan A and matrix metalloprotease1 (MMP1), in spite that the background staining is slightly detected in control bone (Figure 2). These proteins are well-recognized positive markers of melanoma progression. The results indicate that the established melanoma metastasis model accurately reflects the biological events induced by malignant melanoma invasion.

### 2.2. Bone Morphometric Changes Induced by Melanoma Progression

**Figure 3:**
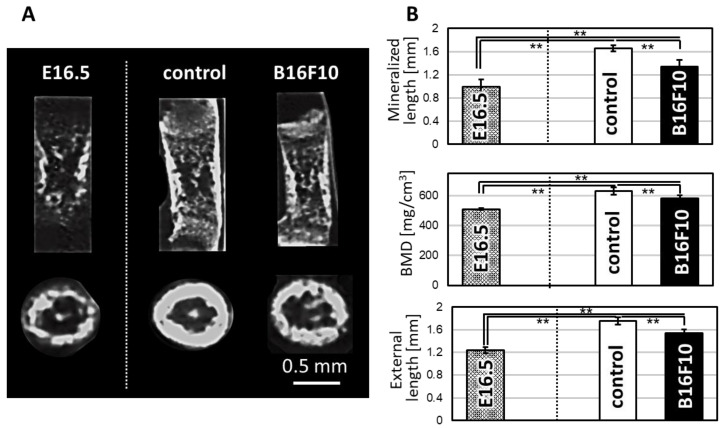
(**A**) μCT X-ray radiographic images of bones before cultivation (E16.5) and after cultivation without cancer cells (control) and with B16F10. (**B**) Bone morphometric analysis including mineralized length, BMD, and external length of the cultured bone. **; *p* < 0.01.

Mice femurs showed increased bone length and BMD (Bone Mineral Density) during cultivation under static loading conditions. On the other hand, coculture with melanoma cells suppressed the longitudinal growth of embryonic femurs compared to control (without cancer cells). Micro-CT images of the horizontal section of femurs indicate increased bone growth on the periosteal side in mouse melanoma B16F10-invaded bone (Figure 3).

### 2.3. Disrupted Organization of Anisotropic Microstructure in Melanoma-Invaded Bone

**Figure 4:**
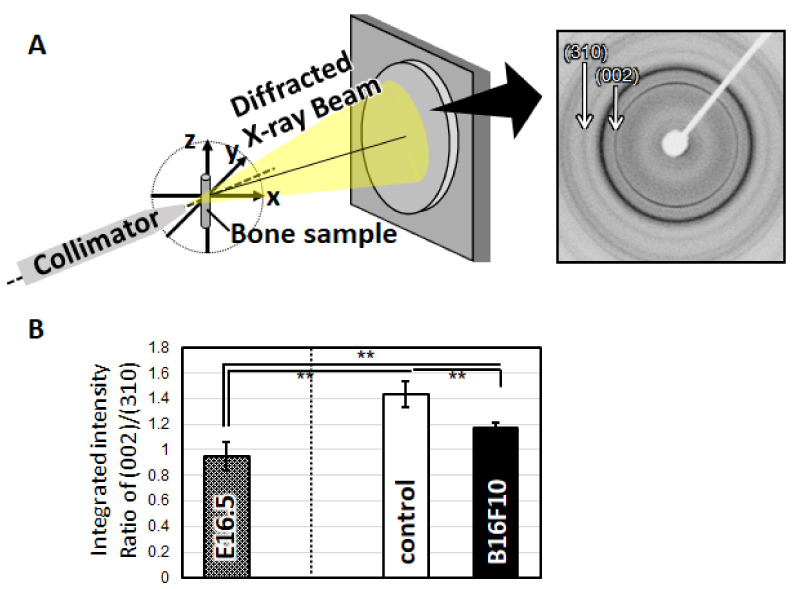
(**A**) Left; schematic illustration of the analysis of apatite orientation using transmission microbeam XRD. Right; μXRD patterns (Debye rings) obtained from the bone sample. Preferential orientation of the *c*-axis of apatite crystals was analyzed with the integrated intensity ratio of (002)/(310). (**B**) Crystallographic orientation of apatite in the longitudinal direction of bone. **: *p* < 0.01.

**Figure 5:**
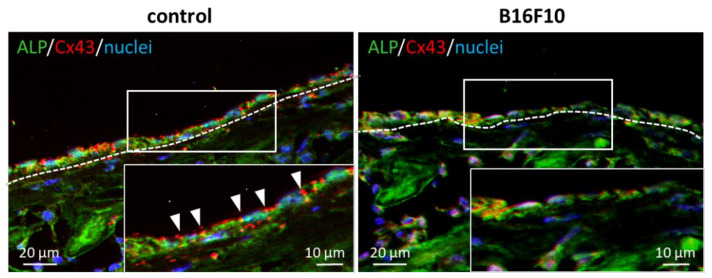
Immunohistochemical images of osteoblast arrangement in cultured bone. Green; ALP, red; Cx43, blue; nuclei. The arrowheads indicate the connexin43-positive region between osteoblasts. The insets show the magnified images of the surrounded area.

Bone tissue cocultured with melanoma cells exhibited a disorganized microstructure with less-aligned apatite crystals (Figure 4). Control bones (without melanoma cells) showed a significantly higher degree of apatite orientation compared to the E16.5 femurs (before cultivation), indicating that the highly organized bone matrix was constructed in response to the external mechanical stimuli. Immunohistochemical analysis of the cultured bone tissue showed that the osteoblasts were arranged in a manner that enabled contact with the neighboring cells on the intact bone surface. On the other hand, the osteoblasts showed isolated, disorganized cell alignment when cocultured with melanoma cells (Figure 5).

## 3. Discussion

Malignant melanoma frequently metastasizes to the bone, resulting in bone dysfunction with increased fracture risk. Homing and progression of melanoma cells in the bone leads to the manifestation of osteolytic pathology via protease activation in melanoma cells, including MMP1 [13]. We have recently observed that the metastasized bone shows disrupted mechanical function derived from the disorganized microstructure of collagen/apatite [9]. One of the most important determinants of the disarrangement of the metastasized bone matrix microstructure is the abnormal alignment of osteoblasts on the bone surface [14]. Furthermore, the dynamic interaction between cancer cells and osteoblasts leads to the development of a randomized matrix structure in cancer-progressed bone tissue [15].

Here, we developed a murine melanoma metastasis model representing the skeletal homing of melanoma using bone tissue cultivation combined with controlled circulating cancer cells. Mouse embryo femurs were cultured under static mechanical loading conditions with stirring of the B16F10 melanoma cells. After cocultivation for 7 d, blackening of the bone tissue was observed, indicating the deposition of melanin on the cultured bone (Figure 1). The results show that murine B16F10 melanoma cells synthesize and deposit melanin, expressing the intact phenotype of melanoma cells even under ex vivo cultivation conditions. Considering that the cultivation environment affects the phenotype of melanoma cells [16], the developed ex vivo metastasis model reflects the progressive melanoma cell activation in vivo. Although two-dimensional experimental models are considered simple to understand, the three-dimensional coculture model unique to melanoma has been desired to elucidate the complex cellular and acellular contributors to metastasis. The characteristic architecture of three-dimensional reconstruct model of bone tissue allows for careful investigation of interaction between melanoma and bone function. Histological analysis showed the homing of MelanA-positive cells in cocultured bone tissue (Figure 2), indicating the successful control of melanoma growth within the bone tissue. Melanoma cells may have invaded the inside of the bone from the periosteal surface with progression during cultivation. However, further histological analysis involving melanoma colonization and invasion distance is necessary to clarify the metastasis features. In addition, the present model utilizes the embryonic mouse bone at the stage before development of the bone marrow center [17]. Considering the clinical scenario of melanoma metastasis, which frequently occurs via vascular, further research using mature bones with well-developed bone marrow is necessary to understand the relationship between melanoma metastasis and bone tissue. However, the proposed model well replicates the periosteal metastasis, which is also recently recognized as an important metastasis niche in melanoma [18]. Homed melanoma cells in bone showed positive expression of MMP1, the expression of which mirrors the invasiveness and the metastatic ability of the melanoma [13]. The bone with metastasized melanoma cells in this study exhibited decreased levels of mineralized length, external length, and BMD (Figure 3). The above-mentioned morphometric alterations are related to the degradation of the extracellular matrix related to MMP1 expression as well as the downregulation of bone formation by controlling osteoblast activities [14]. The effects of soluble molecules derived from melanoma cells can also affect the proliferation and differentiation of osteoblasts, which result in the suppressed bone formation. Furthermore, it is increasingly recognized that osteoclastogenesis is directed by malignant melanoma metastasis [19], the increased bone resorption is possibly related to the osteoclast activation. The metastasis model culturing mature bone tissue with developed bone marrow can allow the understanding of the involvement of osteoclasts in melanoma metastasis. As another factor to consider, oxidative stress acts as a key regulator in metastasis; the recent findings indicate that the increased antioxidant capacity in melanoma cells succeed to control metastasis ability [20].

**Figure 6:**
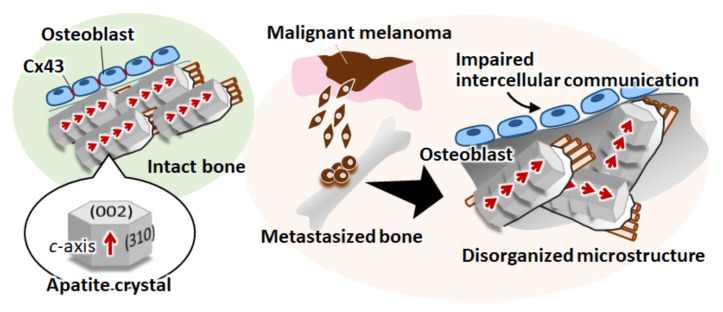
Schematic illustration of the deteriorated bone matrix organization in melanoma-metastasized bone.

The important finding of this study is the disorganization of bone matrix in an ex vivo melanoma metastasis model (Figure 4). The organized bone matrix microstructure is a dominant contributor to bone mechanical function [4]. Understanding microstructural alterations in the affected bone can help in developing therapeutic strategies and biomedical devices for the functional recovery of bone tissue [21,22,23]. For example, the deteriorated balance between osteoblasts and osteoclasts results in mechanical dysfunction owing to the disordered alignment of the collagen/apatite microstructure [24,25,26]. Our recent studies have found that disturbed microstructural alignment in cancer-afflicted bone leads to impairment in metastasized bone, both in cases of osteolytic and osteoblastic metastasis [12,27]. Furthermore, the dynamic interaction between cancer cells and osteoblasts causes the disarrangement of osteoblasts, resulting in a less aligned bone matrix [15]. Indeed, control of osteoblast arrangement in a desired direction is an essential strategy for realization of functionalized bone tissue with controlled anisotropic microstructure [28,29,30]. These studies have contributed to the current understanding of the pathology of bone metastasis and have demonstrated that the molecular interaction between cancer and bone plays a significant role in the determination of bone dysfunction. Particularly, the biological mechanism controlling melanoma-related bone dysfunction has been limited to the understanding of osteolysis regulated by protease activation in melanoma cells [31,32]. Moreover, the involvement of osteocytic arrangement associated with bone matrix microstructure was found to be a potential regulator of melanoma-triggered bone dysfunction [33]. In the present study, the interrupted arrangement of osteoblasts with poor formation of gap junctions, which are positive for connexin43, was found in the B16F10 metastasized bone (Figure 5). Connexin43 plays an important role in osteoblast functionalization by mediating the cytoskeletal organization and the subsequent construction of bone matrix [14,34]. The decreased expression of connexin43 in ALP-positive osteoblasts was found, suggesting the deteriorated interaction between osteoblasts. The impaired expression of connexin43 in osteoblasts likely resulted in a less organized matrix microstructure. Indeed, the aligned osteoblasts produce organized collagen/apatite matrix by controlling the cell-matrix interaction mediated by focal adhesion assembly [30]. Moreover, connexin43 expression in melanoma cells is also a key regulator in metastasis. As indicated in our previous report, the intercellular communication between osteoblast-melanoma cells mediated by connexin43 disturbed the osteoblast alignment [14]. Metastasis related signaling interconnecting osteoblast and melanoma cell can lead to the deteriorated cytoskeletal organization, whereas the intact expression connecting osteoblasts allow the healthy cell alignment. Further experiments are necessary to clarify the connexin43 involvement in metastasis-mediated disruption in cell organization, which will be reported in our future work. Intercellular communication through gap junctions induced the rapid ion exchange as well as sustained physiological response against cancer cells, possibly allowing cytoskeletal disarrangement in the osteoblast. In this study, the disconnection between neighboring osteoblasts resulted in the disorganization of the secreted collagen matrix, leading to the formation of less-aligned apatite crystals in the longitudinal direction of the bone (Figure 6).

## 4. Materials and Methods

### 4.1. Ex Vivo Melanoma Bone Metastasis Model

Mouse melanoma B16F10 cells (ATCC, Manassas, VA, USA) were maintained in DMEM (GIBCO) containing 10% FBS, 100 U/mL penicillin, and 100 μg/mL streptomycin. All cell culture experiments were performed as per the protocols provided in the cell line data sheets. Femurs were obtained from embryonic 16.5-day-old mice (ICR; Japan SLC, Shizuoka, Japan). For the longitudinal organ culture under static loading stress, an ex vivo culturing device was constructed by the combination of cell culture microplates (IWAKI, Tokyo, Japan) and commercially pure (CP) titanium. Each well of the microplate (96 wells) was used for the cultivation of individual bone tissue. To perform uniaxial static loading with a physiological level of force, bone tissue was placed between CP titanium weighing 1.0 g, which possessed hollow spaces for holding the bones between them inside the isolated well. B16F10 cells (2.5 × 10^5^/mL) were circulated around the bones using a magnetic stirrer at approximately 350 rpm. The circulating melanoma cells were allowed to disseminate and arrest at the surface of the bone tissue through the holes placed on the individual culture well for 7 days. For mineralization induction, the bone tissue and cells were incubated in α-modified Eagle’s medium (α-MEM; Gibco, Grand Island, NY, USA) supplemented with 50 µg/mL ascorbic acid (Sigma, St. Louis, MO, USA), 10 mM β-glycerophosphate (Tokyo Kasei, Tokyo, Japan), and 50 nM dexamethasone (MP Bioscience, Solon, OH, USA) at final concentrations at 37 °C in 5% CO_2_, and the medium was changed after 3 days. The experiments were designed with the number of replications of *n* = 5. All animal experiments were approved by the Osaka University Committee for Animal Experimentation (approval number: 27-2-1). All experiments were performed in accordance with the related guidelines and regulations for scientific and ethical animal experimentation.

### 4.2. Bone Morphology Analysis

Femurs were scanned using micro-CT (Shimadzu, Kyoto, Japan) at X-ray energy settings of 25 kV and 130 μA, with a nominal resolution of 10 μm. Bone mineral density (BMD) was quantitatively analyzed based on micro-CT images. The calibration curve of CT value-BMD was prepared using phantoms, which contain hydroxyapatite in the range 200–800 kg/m^3^. The CT value was subsequently converted to BMD values using the TRI-3D BON software (RATOC System Engineering, Tokyo, Japan).

### 4.3. Analysis of Apatite Orientation

The degree of apatite orientation was analyzed using a microbeam X-ray diffractometer (μXRD) system (R-Axis BQ, Rigaku, Tokyo, Japan) equipped with a transmission optical system (Mo-Kα radiation). The incident beam was radiated vertically to the long axis of the bone at a tube voltage of 50 kV and a tube current of 90 mA. The degree of preferential orientation of the c-axis in the apatite crystals was determined as the relative intensity ratio of the (002) diffraction peak to the (310) peak in the X-ray profile. This was previously reported as a suitable index for the evaluation of apatite orientation [4,5].

### 4.4. Immunohistochemistry

The cultured femurs were fixed in neutral buffered formaldehyde for 24 h, followed by decalcification using 0.5 M EDTA-2Na solution (pH 7.4) for 7 d. Specimens were dehydrated through a graded series of ethanol, embedded into paraffin, and transversely cut into 5 μm-thick sections. Deparaffinized sections were incubated with normal goat serum (Thermo Fisher Scientific, Waltham, MA, USA) to block non-specific antibody binding sites. The specimens were then incubated with rabbit anti MMP1 (Abcam, Cambridge, UK), mouse anti melanA (Santa Cruz, Dallas, TX, USA), mouse anti ALP (Novus Biologicals, USA, Centennial, CO, USA), and rabbit anti Cx43 (Cell Signaling, Danvers, MA, USA) antibodies. The secondary antibodies used were as follows: Alexa Fluor 546-conjugated anti-rabbit IgG (Molecular Probes, Thermo Fisher Scientific, Waltham, MA, USA), and Alexa Fluor 488-conjugated anti-mouse IgG (Molecular Probes, Thermo Fisher Scientific, Waltham, MA, USA). Nuclei were stained with DAPI (Thermo Fisher Scientific, Waltham, MA, USA).

### 4.5. Statistical Analysis

Statistical significance was assessed using one-way ANOVA, followed by Tukey’s post hoc test. A significance of *p* < 0.05 was considered for rejection of the null hypothesis.

## 5. Conclusions

Skeletal metastasis of malignant melanoma causes severe bone tissue dysfunction. Here, we found that the disorganized alignment of apatite crystals in bone matrix was associated with the homing of melanoma cells; this was assessed by developing an artificially controlled ex vivo bone metastasis model. Furthermore, the disorganized alignment of osteoblasts on the melanoma-metastasized bone in relation to the less aligned bone matrix microstructure was observed. Our findings can help provide novel therapeutic targets for the recovery of the mechanical function of bone tissue by controlling the organization of bone matrix alignment by regulating osteoblast arrangement.

## Data Availability

The data presented in this study are available on request from the corresponding author.
